# Optimizing Treatment of Infective Endocarditis in Patients Who Inject Drugs

**DOI:** 10.3390/antibiotics15090881

**Published:** 2026-09-09

**Authors:** Jarett Worden, Tyler Baumeister, Ellen Eaton

**Affiliations:** 1 College of Pharmacy & Health Sciences, Belmont University, Nashville, TN 37212, USA; 2 Pharmacy, Williamson Medical Center, Franklin, TN 37067, USA; tbaumeister@williamsonhealth.org; 3 Department of Psychiatry and Behavioral Neurobiology, University of Alabama at Birmingham, Birmingham, AL 35233, USA; eeaton@uabmc.edu

**Keywords:** endocarditis, persons who inject drugs, PWID, intravenous drug use, substance use disorder, *Staphylococcus aureus*, outpatient parenteral antibiotic therapy, OPAT, long-acting lipoglycopeptides, oral antibiotics

## Abstract

The treatment of infective endocarditis (IE) in patients who inject drugs (PWID) is complex and challenging. These patients face stigma in the healthcare system, as well as higher mortality, longer length of hospital stay, higher rates of recurrent infections, and lower likelihood of antibiotic completion. Standard treatment for IE involves several weeks of antibiotics. For PWID, these prolonged courses often mean intravenous antibiotics administered in the inpatient setting for at least 4 to 6 weeks, which increases both healthcare costs and the risk of patient-directed discharge against medical advice. Effective treatment of IE in PWID involves a combination of addiction management and appropriate antibiotic therapy. To promote rational use of healthcare resources and optimize antibiotic stewardship, novel approaches to antibiotic therapy must be utilized. This review discusses evidence supporting treatment strategies outside of the inpatient hospital setting, including outpatient parenteral antibiotic therapy (OPAT), long-acting lipoglycopeptides, and oral antibiotic therapy for the treatment of endocarditis. Optimal treatment of IE in PWID involves support from a multidisciplinary healthcare team, management of substance use disorder, and effective antibiotic therapy.

## 1. Introduction

Infective endocarditis (IE) is a severe infection associated with significant morbidity, mortality, prolonged hospital stays, and increased healthcare costs [1]. Over the last few decades, the epidemiology of IE has shifted along with the ongoing opioid and substance use epidemics. This has resulted in increased hospitalization and mortality due to IE among persons who inject drugs (PWID) [2,3]. Injection drug use continues to be one of the main risk factors for IE in the United States, particularly among young adults who lack predisposing cardiac conditions. The management of IE in PWID poses significant challenges for antimicrobial stewardship programs for many reasons. Standard of care involves prolonged intravenous (IV) antibiotic therapy managed by a multidisciplinary team often involving infectious diseases specialists, pharmacists, social workers, and cardiothoracic surgeons. Treatment is further complicated by social issues such as unstable housing, psychiatric comorbidities, and limited healthcare and resource access. All of these factors can lead to potential delayed diagnosis, prolonged hospitalization, premature hospital discharge, and increased risk of reinfection.

Antimicrobial stewardship programs continue to balance practicing evidence-based medicine while considering patient-centered approaches that address these barriers. Novel strategies for treatment such as outpatient parenteral antibiotic therapy (OPAT), long-acting lipoglycopeptides, and oral stepdown therapy offer opportunities to improve outcomes in these patients while reducing utilization of healthcare resources [4]. This narrative review discusses the unique epidemiology and risk factors for PWID, stigma surrounding substance use disorders, and strategies to improve care and optimize antibiotic utilization in this vulnerable population.

## 2. Challenges for Patients Who Inject Drugs

### 2.1. Risk Factors for Patients Who Inject Drugs

PWID are at an increased risk of IE and other severe infections due to a combination of behavior-, social-, microbiological-, and healthcare-related factors. The traditional IE population involves patients with valvular disease and frequent healthcare exposure, while PWID often develop infection through repeated introduction of microorganisms into the bloodstream during the injection process. Non-sterile injection techniques, sharing or reusing needles, contaminated water, and illicit drug preparations facilitate direct inoculation of bacteria into the bloodstream.

*Staphylococcus aureus* remains the predominant pathogen causing IE among PWID [4]. Colonization with *S. aureus* is common in this population due to frequent skin trauma, poor hygiene, and recurrent healthcare exposure due to overdose. *S. aureus* bacteremia is associated with up to a 30% one-year mortality rate [5]. Independent predictors of mortality for IE include older age, acute heart failure, and discharge against medical advice [6]. Other pathogens may also occur, such as *Streptococcus*, *Enterococcus*, Gram-negative organisms, and *Candida* species, especially in patients with repeated healthcare exposure or non-sterile injection techniques [1]. Patients with repeated episodes of bacteremia are at risk for developing endothelial damage and valvular colonization.

Anatomically, right-sided IE is more common among the PWID population compared to the general public [4]. Studies continue to show that PWID face additional morbidity and mortality with IE compared to the general population. Compared to patients who do not inject drugs, PWID have a 16-times greater risk of developing invasive methicillin-resistant *Staphylococcus aureus* (MRSA) infections [7]. While overall mortality related to endocarditis has decreased, younger patients with substance use disorders are seeing increases in mortality [8]. PWID also have higher rates of bacteremia with cardiac involvement, longer lengths of hospital stay, lower rates of antibiotic completion, and higher rates of recurrent endocarditis [9,10,11]. Prior episodes of IE greatly increase the risk for recurrence along with common concurrent infections such as skin and soft tissue infection, osteomyelitis, and septic thrombophlebitis. This creates a cycle of repeated infection, hospitalization, and antibiotic exposure, all contributing to poor outcomes. Patients with prosthetic valves, intracardiac devices, or underlying valvular abnormalities are at an even greater risk when combined with injection drug use. Recurrence represents a major challenge in this population that is driven by ongoing substance use following completion of antibiotic therapy (or incomplete antibiotic therapy). From an infectious disease treatment perspective, successful treatment is not only dependent on antibiotic therapy but also strategies to mitigate substance abuse, thereby reducing risks for reinfection and improving long-term outcomes.

### 2.2. Stigma as Barrier to Care

Stigmas related to substance use disorders (SUD), and specifically injection drug use, are more pervasive than stigmas towards other vulnerable groups, including persons with HIV and gender and sexual minorities [12]. SUD stigma can have dangerous public health impacts leading to delays in addiction care, barriers to retention in care, and return to use [13]. Stigma affects health outcomes beyond addiction too: SUD stigma from both community and healthcare workers can deter patients who use drugs from seeking medical care for life threatening conditions like infections (i.e., HIV, endocarditis) [14]. Unfortunately, fear of SUD stigma, untreated pain, and withdrawal causes patients to present for medical attention later with more advanced illness, including serious injection-related infections like endocarditis [15]. Delayed care can lead to devastating results when it comes to serious injection-related infections, which are fatal in up to 10% of cases [16]. For patients who do seek care, the use of non-stigmatizing language documented in health records has been associated with improved health outcomes, suggesting that provider stigma (or lack thereof) may be present both in documentation and clinical care delivery [17].

Stigma can be mitigated through the education of healthcare workers, staff, and administrators. One study found that a brief, one hour on-demand training on SUD as a chronic disease was associated with stigma reductions among healthcare workers [18]. Another way to reduce stigma is to incorporate staff and clinicians with lived experience in addiction and recovery, also known as peer recovery coaches. By incorporating staff with lived experience, health systems can work to reduce stigma from the inside out and present a healing, patient-centered experience to those with SUD.

### 2.3. Source Control: Mental Health Counseling and Addiction Management in Conjunction with Antimicrobial Treatment

Effective source control for serious infections among people with SUD requires addressing the underlying addiction and mental health (MH) comorbidities: a comprehensive approach that integrates anti-infective treatment with mental health treatment and addiction management. This may involve pharmacotherapy, cognitive behavioral therapy, and/or peer support. Research highlights that addressing the underlying substance use and co-occurring MH conditions is essential to improving clinic and treatment adherence, reducing reinfection risk, and achieving successful clinical outcomes [19,20]. In fact, a recent report by the National Academy of Medicine found that integrated programs that combine care for SUD, along with mental illness, with infectious disease prevention and care is best practice [21].

For some serious injection-related infections, such as endocarditis and bloodstream infections, inpatient care is the only way to provide life-saving antibiotics, surgery, and specialty care [22]. Yet, when SUD and mental illness are neglected and/or stigma compromises the therapeutic environment, patients may leave the inpatient setting prematurely, also known as patient-directed discharge (PDD). This poses a significant challenge, as premature discontinuation of care can lead to incomplete treatment, recurrent infection, rehospitalization, and increased morbidity [16]. Recent data highlight the benefit of oral antibiotics (versus no antibiotics) at the time of discharge for patients who leave the hospital prematurely. In one study, oral antibiotics at the time of premature discharge led to a significant reduction in 90-day readmission for persons with serious injection-related bacterial infections [23]. In this single site study, the readmission rate was 69% for those who did not receive oral antibiotics compared with 33% among those who received partial course of oral antibiotics at the time of premature discharge. A retrospective cohort analysis of patients with *S. aureus* bacteremia compared patients who either received (1) the full IV antibiotic course inpatient, (2) partial IV antibiotics followed by discharge with oral antibiotics, or (3) partial IV antibiotics followed by discharge without oral antibiotics (incomplete therapy) [24]. Microbiologic treatment failure was higher in the patients who received incomplete therapy compared to patients who received either complete IV therapy or partial oral therapy (44.4% vs. 10.7% vs. 13%). These studies led to a paradigm shift for the care of serious injection-related infections as they highlight the notion that oral antibiotic therapy at discharge may offer a pragmatic and patient-centered alternative for selected individuals, improving treatment completion while reducing the need for prolonged hospitalization and accommodating patients’ preferences and life circumstances.

Stigma, SUD, and co-occurring mental illness serve as reversible barriers to addiction treatment and, ultimately, infection treatment and prevention. Working across healthcare systems to educate clinicians on SUD as a chronic and treatable disease may reduce stigma among healthcare providers and increase access and engagement for persons who use drugs. Addiction and mental health conditions can and should be treated in persons who use drugs to help them adhere to antimicrobial and other disease management and prevent future infections including bacterial and viral diseases. Integrating services including care for infections, SUD and mental health conditions reduces barriers for patients and is best practice.

### 2.4. Surgical Intervention

In addition to management of SUD, PWID may also require surgical intervention to manage IE, especially patients with risk of embolic events, abscess, or heart failure. Compared to patients that are not addicted to drugs, PWID have higher risks of relapse infections, recurrent infections, and one-year post-operative mortality [25]. Collaboration with cardiothoracic surgery is an essential component of multidisciplinary care. A recent survey of healthcare facilities in Tennessee found that 86% of the facilities that performed cardiac surgery indicated they “sometimes” perform valve surgery on patients with native valve endocarditis in PWID [26]. That number decreased by half for PWID who had previous valve replacement surgery. Patients undergoing invasive cardiac surgery may have longer recovery time and longer hospitalizations. For right-sided endocarditis, catheter-based interventions may be a less invasive option for the removal of tricuspid valve vegetations, but this will not remove all infected tissue. While surgery is an important consideration in the treatment of IE, a comprehensive review of cardiac surgery is outside of the scope of this review.

## 3. Optimizing Antimicrobial Treatment in PWID

The treatment of infective endocarditis traditionally requires prolonged IV antimicrobial therapy often lasting 4–6 weeks. This approach remains the standard of care; however, barriers such as unstable housing, limited access to outpatient services, concerns for the misuse of indwelling catheters, and PDD are all challenges faced by the healthcare team when caring for these patients. These challenges have sparked interest in alternative treatment strategies to improve outcomes while maintaining clinical effectiveness.

It is important to evaluate patient specific factors and preferences when selecting alternative treatment options (Table 1). Each therapeutic alternative has potential advantages and disadvantages, and not all patients would be good candidates for deviations from standard-of-care antibiotic therapy. Much of the supporting evidence for these strategies come from smaller studies, including retrospective and observational studies. When considering alternative options, clinicians should weigh the level of evidence, limitations, and patient populations studied in the literature (Table 2). Clinicians should also assess the resources available to the patient, as well as the monitoring and follow-up required for successful treatment.

### 3.1. OPAT as an Option: Outpatient Parenteral Antibiotic Therapy

For many patients with prolonged courses of IV antibiotics, OPAT is a potential option that allows patients to complete antibiotic therapy outside of the hospital. Patients are typically discharged with a central venous catheter, such as a peripheral inserted central catheter (PICC), to receive IV antibiotics either at home or at skilled nursing facilities. However, patients with a history of SUD are often excluded from this option due to concern that the patient may access the central line for illicit drug use. This begs the question: can OPAT be utilized safely and successfully in PWID?

A review of 10 studies that evaluated OPAT in 800 PWID found that there were no differences in completion of therapy, mortality, or adverse events related to catheters when compared to patients who do not use injectable drugs, although readmission rates may be higher in PWID [27]. Interestingly, the studies that evaluated misuse of PICC lines found very low rates of tampering or misuse among these patients (≤2%). Perhaps the better question is not *if* but *how* OPAT can be used effectively and safely in PWID.

Several recent studies have examined addiction treatment in combination with OPAT. One small, pilot, randomized trial compared patients with SUD who were randomized to either inpatient IV antibiotic completion or OPAT [28]. All patients received treatment for SUD by an addiction management physician, consultation with an infectious disease provider, had follow-up appointments with an outpatient physician (at least weekly), and received buprenorphine therapy in-hospital and at discharge. Hospital length of stay in the OPAT group was half that compared to usual care (22.4 days vs. 45.9 days). Preliminary results of the larger Buprenorphine plus Outpatient Parenteral Antibiotic Therapy (BOPAT) trial were presented at ID Week 2025 and appeared promising [29]. Compared to standard of care, buprenorphine plus OPAT resulted in significantly shorter hospital length of stay and less antibiotic days missed, as well as fewer serious adverse drug reactions for patients with serious injection-related infections. In another small, retrospective study of 20 patients with severe infections that met criteria for OPAT and buprenorphine therapy, there were no deaths, overdoses, or central line complications reported [30]. The 30-day readmission rates were similar between patients discharged with OPAT and those who were not. Patients had to have safe housing, abstinence from illicit substance use, and agree to return for follow-up appointments to qualify for OPAT. While three patients did relapse and injected drugs during OPAT, not a single patient used the PICC line as an injection site. It is important to note that many of these studies are small with strict inclusion criteria and predisposition for selection bias, highlighting the importance of appropriate patient selection and resources for OPAT. Not all studies use standardized metrics of housing stability, treatment engagement and/or continued substance use or recovery for patient inclusion, which limits generalizability. A summary of patient selection and special considerations for OPAT is provided in Table 1.

### 3.2. POETs and Pills: Oral Antibiotic Therapy

Oral antibiotic therapy is another approach to severe infections in PWID that is gaining momentum. Since the publication of the Partial Oral Treatment of Endocarditis (POET) trial, practitioners have become more open-minded about using oral antibiotics for endocarditis. The study showed no difference between partial oral versus IV antibiotic therapy in the composite outcome of all-cause mortality, unplanned cardiac surgery, embolic events, or relapse of bacteremia [31]. All patients received at least 10 days of IV antibiotic therapy before transitioning to oral therapy and 80% of patients in the oral treatment arm were at least partially treated in the outpatient setting. Almost 40% of patients in each arm underwent valve surgery during hospitalization. Although the POET trial was a large, well-designed, randomized, multicenter trial, many have struggled to connect these results to PWID due to several limitations. First, only patients with left-sided endocarditis were included, but the majority of PWID develop right-sided endocarditis. Second, only 1% of patients had a history of IV drug use. Less than a quarter of patients treated with oral antibiotics in this trial were infected with *S. aureus*, and zero patients had MRSA. Lastly, the oral antibiotic regimens used were complex and may not be widely applicable, although adverse effects were minimal in the trial. While the POET trial was a landmark study, the more recent real-world work by Freling et al. may be more clinically meaningful for PWID [32]. Although this multicenter cohort study was retrospective and had fewer patients (46 patients received oral stepdown), it more closely represented the epidemiology of endocarditis seen in PWID. Like the POET trial, this study included patients with infective endocarditis treated with either IV or oral stepdown antibiotics (after a median of 15.5 days of IV therapy). Compared to the POET trial, this study had higher proportions of right-sided endocarditis (35% vs. 0), more patients with a history of IV drug use (37% vs. 1%), and higher rates of *S. aureus* (63% vs. 22%) and MRSA (35% vs. 0). The most common antibiotic regimens contained linezolid, whereas the POET trial primarily used either dicloxacillin or amoxicillin plus rifampicin for most patients with *S. aureus*. No significant differences were noted between the two groups for 90-day clinical success, mortality, recurrence of infection, or hospital readmission. There were significantly fewer adverse drug events reported in the oral group compared to the IV group (8.7% vs. 27.5%), including acute kidney injury and line-related events.

Another single-center retrospective study of IE therapy with a large number of PWID found similar results when patients were transitioned to oral antibiotic therapy. There was no difference in 90-day all-cause mortality and 90-day relapse of infection between full IV antibiotic therapy and partial oral antibiotics [6]. A retrospective cohort analysis also found no difference in microbiologic treatment failure outcomes between full IV antibiotic inpatient courses and partial IV antibiotics with oral antibiotics at discharge in PWID with complicated *S. aureus* bacteremia [24].

Despite the mounting evidence for safety and efficacy of partial oral antibiotic therapy in patients with severe infections, providers still appear to remain hesitant. A recent survey of adult infectious diseases providers indicated that most prescribers never or rarely use partial oral therapy for endocarditis, while only 10% of providers indicated they use oral antibiotics in >25% of patients [33]. Those numbers appear higher for PWID, with 34% of physicians indicating they often consider partial oral regimens in these cases. Barriers to prescribing oral therapy included fear of treatment failure, adherence, lack of current evidence, patient comorbidities, and legal concerns. In summary, transition to oral antibiotic therapy appears to be safe and effective in patients who are clinically stable, have adequate IV therapy lead-in, can tolerate multiple oral antibiotics, and have a history of medication compliance.

### 3.3. Connecting the DOTS: Long-Acting Lipoglycopeptides

Dalbavancin and oritavancin are long-acting lipoglycopeptides with potent activity against Gram-positive organisms, including methicillin-susceptible *S. aureus* and MRSA. Therapeutic drug concentrations persist for weeks after administration due to prolonged elimination half-life, allowing for treatment with one or two doses rather than daily administration [34,35]. This eliminates the need for prolonged central venous access, which is ideal for populations such as PWID.

One of the most notable trials with evidence supporting dalbavancin comes from the Dalbavancin as an Option for Treatment of *S. aureus* Bacteremia (DOTS) trial [36]. This multicenter, randomized control trial enrolled patients with complicated *S. aureus* bacteremia, including those with definite or possible right-sided infective endocarditis (5%), after initial clearance of bacteremia with standard IV therapy. Participants were randomized to receive either two doses of IV dalbavancin 1500 mg (separated by one week) for completion of therapy or continued standard IV treatment. The results demonstrated that dalbavancin achieved outcomes comparable to standard IV therapy, with similar treatment success rates and safety profiles. Although superiority was not demonstrated, it established that dalbavancin was noninferior to conventional therapy for the completion of treatment in appropriately selected patients. While these results may not support a change to the current standard of care, the findings support dalbavancin as a viable treatment strategy following initial clinical stabilization and bloodstream clearance. From a stewardship perspective this offers a potentially effective means to deliver antibiotic therapy while reducing hospitalization, catheter-related complications, OPAT utilization, and healthcare resources.

More recent evidence also supports a potential role for long-acting lipoglycopeptides beyond dalbavancin, although the available data remain limited. A 2025 retrospective cohort of 27 patients were treated with oritavancin for *S. aureus* bacteremia, with 59% of those being PWID. Patients received a mean of 10 days of conventional IV therapy before transition to oritavancin, achieving a 96% clinical cure rate, 89% survival at 180 days, and an average reduction of 18 inpatient hospital days per patient. Notably, 89% of patients demonstrated a positive return on investment, suggesting that long-acting agents may provide substantial clinical and economic benefits for PWID who otherwise require prolonged hospitalization because of barriers to OPAT [37]. However, the small non-comparative cohort consisted of patients who had generally achieved clinical stability before oritavancin administration, introducing potential for selection bias. The use of oritavancin for more serious infections like bacteremia and endocarditis remains off-label, and its acquisition cost, limited ability to modify therapy after administration, potential effects on resistance and antimicrobial stewardship all must be considered. Additionally, recurrence or loss to follow-up may be difficult to assess in PWID. Therefore, these findings should be hypothesis-generating, and prospective comparative studies are needed to define safety, effectiveness, economic value and ideal patient selection when considering oritavancin therapy for invasive *S. aureus* infections.

Other observational studies have consistently identified PWID as a population likely to benefit from long-acting antimicrobial strategies. In one retrospective evaluation of PWID with serious *S. aureus* infections (including IE), dalbavancin was used as completion therapy after an initial course of conventional IV antibiotics. Clinical response rates were favorable among patients who completed the intended regimen, supporting the feasibility of this approach in patients with significant barriers to standard treatment [38]. Additional multicenter observational studies and systematic reviews have reported encouraging outcomes with dalbavancin as sequential or consolidation therapy for infective endocarditis. Across published cohorts, clinical failure rates were generally low, safety was favorable, and the most common reasons for selecting dalbavancin included inability to safely administer OPAT, anticipated nonadherence, and active substance use. Several reports specifically highlight PWID as a population in whom dalbavancin may improve treatment completion while reducing the risks associated with prolonged IV access [39,40].

**Table 2:** Summary descriptions of included studies.

Study	Study Design	Number of Patients	Infection Type	PWID %	Discharge Antimicrobial Regimen	Limitations
**Outpatient Parenteral Therapy**						
Fanucchi et al. [28]	Pilot randomized trial	20	SIRI (IE: 90%)	100%	OPAT discharged home when medically stable. Specific antibiotics not reported. OPAT patients received SUD assessment by addiction medicine physician, management by ID physician, home antibiotic deliveries, and dressing changes.	Small sample size; single center design; intensive outpatient follow-up and interventions.
Price et al. [30]	Retrospective chart review	68	Various (Bacteremia/IE: 73.5%)	100%	OPAT via PICC line discharged home. Included patients had safe housing, support, engagement with addiction treatment, abstinence from illicit substances prior to discharge, and agreed to follow-up care. OPAT patients received care at a local rapid access, low barrier, multidisciplinary SUD clinic.	Retrospective study; small sample size; single center; OPAT patients were more likely to have been on MOUD already; outcomes for non-OPAT patients not reported.
Beieler et al. [41]	Retrospective chart review	53	Various (IE: 28%)	53% (current IDU)	OPAT administered via PICC line at medical respite (with one to two nursing visits per day). Patients were provided resources for addiction treatment, MOUD, and harm reduction strategies.	Retrospective study; small sample size; single center; included only patients experiencing homelessness.
Jewell et al. [42]	Retrospective chat review	205	Various (IE: 16.5%)	100%	OPAT administered via PICC line at residential addiction facility.	Retrospective study; single center; lack of comparator data.
**Partial oral antibiotics**						
Iversen et al. [31]	Randomized, unblinded trial	400	IE	1%	Partial oral therapy after at least 10 days of IV therapy. Multiple different oral antibiotic combinations used; most common antibiotic regimens for *S. aureus* IE were dicloxacillin plus rifampicin (33%), amoxicillin plus rifampicin (29%), and moxifloxacin plus rifampicin (7%).	Included only left-sided IE; very small percentage of PWID; low incidence of *S. aureus* IE (no MRSA).
Freling et al. [32]	Retrospective, multicenter cohort	257	IE	37%	Multiple different oral antibiotics; most common was oral linezolid 600 mg twice daily, alone or in combination with rifampin (65%). The median duration of IV lead-in treatment was 15.5 days for the oral cohort.	Retrospective study; readmission to other hospitals may not be captured.
Wildenthal et al. [24]	Retrospective cohort	238	*S. aureus* bacteremia (IE: 64.7%)	100%	Patients received either SOC IV antibiotics (during inpatient admission), incomplete IV antibiotic therapy (with no oral antibiotics), or transition to partial oral antibiotic therapy at discharge after at least 10 days of IV antibiotics. The median duration of IV antibiotics before transition to oral antibiotic therapy was 18 days. Most patients had addiction medicine consultation (70%) and over half of the patients received MOUD.	Retrospective study; single center; higher loss to follow-up in patients discharged with incomplete therapy (may underestimate of risk of death); patients who died prior to discharge were excluded (favoring SOC cohort).
El-Dalati et al. [6]	Retrospective cohort	236	IE	58% had recent IDU (within the last 30 days)	Patients received at least 10 days of IV antibiotics or 7 days from valve surgery. Oral linezolid was the most common antibiotic for *S. aureus* IE. The median duration of IV lead-in treatment was 18 days for the oral cohort. All patients with active SUD or a history of IDU were offered addiction medicine consultation.	Retrospective study; single center; cohorts were not matched; valve surgery was more common in the oral antibiotic cohort.
**Long-acting lipoglycopeptides**						
Morrisette et al. [39]	Retrospective chart review	56	Various (IE: 23%)	30%	Patients received an undisclosed amount of IV antibiotic therapy before discharging on dalbavancin (71%) or oritavancin (25%) for 1–2 doses. Of note, concomitant oral antibiotics during or after the long-acting lipoglycopeptide were prescribed in 29% of the PWID group and 31% in the non-PWID group.	Retrospective, nonrandomized design; small PWID sample size; heterogeneous infections and treatment; many patients lost to follow-up.
Hidalgo-Tenoria et al. [40]	Multicenter, observational retrospective cohort	83	Bloodstream infections (IE: 40.9%)	Not specified	Patients received various antibiotics before administration of dalbavancin. Dosing regimens ranged from 1000 to 1500 mg on day one as a single dose or multiple doses. The repeat doses (500–1000 mg) were given every 7–15 days as deemed appropriate depending on anticipated remaining duration of treatment.	Retrospective observational design; selection bias for patients who had clinically stabilized; heterogeneous dosing regimens; no specified PWID population.
Turner et al. [36]	Multicenter, randomized, open-label trial	200	Various (right-sided IE: 5%)	15%	Patients received dalbavancin 1500 mg follow by another dose of 1500 mg a week later. Patients had to ≥72 h but not more than 10 days of effective initial antibiotic therapy before randomization	Open label design; small PWID group; left-sided IE was excluded.
Jordan et al. [37]	Retrospective, single arm observational cohort	27	*S. aureus* bacteremia (IE: 26%)	59%	A proportion of 89% of patients received oritavancin 1200 mg × 1 dose and 11% received 1200 mg × 2 doses (without a known interval in between). Patients received a mean duration of 10 days ± 8 days of IV antibiotics before oritavancin. 44% of patients did receive additional oral antibiotics after oritavancin.	Small sample size; retrospective, observational design; no comparator group; almost half received additional oral therapy; heterogeneous infections and oritavancin regimens.

Abbreviation: ID, infectious diseases; IDU, intravenous drug use; IE, infectious endocarditis; IV, intravenous; MOUD, medication for opioid use disorder; MRSA, methicillin-resistant *Staphylococcus aureus*; OPAT, outpatient parenteral antibiotic therapy; PICC, peripheral inserted central catheter; PWID, persons who inject drugs; SIRI, severe injection-related infections; SOC, standard of care; SUD, substance use disorder.

### 3.4. Optimization of Healthcare Resources

The management of IE in PWID results in substantial healthcare costs, including prolonged hospitalizations, frequent readmissions, and significant coordination of care from the healthcare team. Social challenges in PWID such as unstable housing, concern for central venous catheter misuse, and lack of social support can make completing the standard 4–6 weeks of antibiotic therapy outpatient impractical. Antimicrobial stewardship programs play a large role in supporting strategies to maintain clinical effectiveness, ensure treatment completion and ensure patient safety, while reducing healthcare utilization.

Hospital length of stay (LOS) is one of the biggest cost burdens associated with managing endocarditis and other severe infections in PWID. Long-acting lipoglycopeptides such as dalbavancin offer a unique and attractive stewardship opportunity as they provide weeks of antimicrobial coverage through one or two infusions, helping to facilitate earlier hospital discharge. Multiple observational studies have demonstrated reductions in hospital LOS among patients transitioned to dalbavancin compared with conventional IV therapy. In a retrospective comparison of serious Gram-positive infections, including infective endocarditis, patients receiving dalbavancin experienced significantly shorter hospital stays than those receiving standard-of-care therapy (5.2 vs. 7.2 days; *p* = 0.01) without increased treatment failure or readmission rates [43].

Although traditionally long-acting lipoglycopeptides have been associated with high acquisition cost, recent generic availability of dalbavancin has reduced these cost implications. Even at a high cost, factors such as hospital bed utilization, nursing resources, vascular access placement, and laboratory monitoring all contribute substantially more to treatment costs compared to the drug itself. A pharmacoeconomic analysis derived from the REDS study demonstrated that dalbavancin use was associated with substantial reductions in healthcare costs compared with conventional IV glycopeptide therapy [44]. Among 170 patients with acute bacterial skin and skin structure infections, those treated with dalbavancin had shorter overall hospitalizations (11.3 vs. 15.8 days) and markedly shorter post-treatment lengths of stay (7.8 vs. 14.1 days). Despite higher antibiotic cost, reductions in hospitalization translated into a 28.5% decrease in total hospitalization costs and a 44.2% reduction in post-treatment hospitalization costs.

Studies have also demonstrated overall decreased costs when utilizing OPAT as a treatment strategy for severe infections in PWID. Patients with SUD treated with OPAT at a medical respite demonstrated institutional cost savings of $25,000 per episode [41]. Another retrospective analysis of PWID treated with OPAT in a residential addiction treatment center showed cost savings of over $2 million over a six-year period, saving over $11,000 per episode of OPAT [42]. A recent microsimulation modeling study compared standard of care, OPAT, and partial oral antibiotics strategies for treating severe infections in PWID. The simulation found that the OPAT strategy was the least expensive option, whereas standard of care was the most expensive [45].

It should be emphasized that these strategies and earlier hospital discharge may not be appropriate for every PWID. Patients with active SUD often have poor social support, lack access to stable housing, have uncontrolled MH comorbidities, lack of follow-up addiction and medical care, and may be underinsured or uninsured. Proper planning for both inpatient and outpatient care and follow-up by a multidisciplinary team that includes mental health professionals and social workers are essential for the successful implementation of the strategies. Premature discharges without appropriate resources for simply monetary savings could result in worse outcomes and hospital readmission, costing the health system more.

## 4. Conclusions

Optimized treatment of IE in PWID involves a multidisciplinary team approach that includes both antimicrobial therapy and addiction management. Stigma surrounding SUD may impair care and serve as a barrier to addiction treatment. There is growing evidence to support effective antimicrobial treatment outside of the hospital setting, including OPAT, long-acting lipoglycopeptides, and oral antibiotic therapy as alternative treatment options in select patients. Because of long durations of IV antibiotics, burden of healthcare resources, and emerging safety and efficacy data of these alternative therapies in PWID, clinicians should consider these as potential options for appropriately selected patients.

## Figures and Tables

**Table 1:** Summary of infective endocarditis treatment strategies for patients who inject drugs.

	Outpatient Parenteral Antibiotic Therapy	Oral Antibiotics	Long-Acting Lipoglycopeptides
Patient Selection	Stable housing for home OPAT Reliable social support and transportation Active treatment/management of SUD Access to home health or skilled nursing facility	Received at least 2 weeks of IV antibiotics Do not have access to home health or skilled nursing facility Able to tolerate multiple pills administered multiple times per day History of compliance with oral medications Patient-directed discharge	Non-compliant patients Reliable social support and transportation (if follow-up doses required) Do not have access to home health or skilled nursing facility Good candidate for peripheral line access (if needed for follow-up doses)
Advantages	Scheduled follow-up with healthcare liaisons Opportunity to provide addiction care Opportunity for laboratory monitoring Reduced hospital length of stay Reduced cost	Does not require PICC or IV therapy outpatient Does not require skilled nursing facility or home health Reduced hospital length of stay Reduced cost	Only one or two doses Does not require PICC Does not require skilled nursing facility or home health Reduced hospital length of stay Reduced cost
Disadvantages	Line associated complications/infections Requires stables housing, social support, and transportation Often requires insurance	Potential for drug–drug interactions (especially with rifamycins) Loss to follow-up Cost of antibiotics (especially without insurance)	May have loss to follow-up due to lack of need for continued therapy Requires clinic/nursing coordination IV access for each administration Medication cost is expensive
Barriers to Implementation:			
Lack of adequate social support Lack of stable housing Unwilling to commit to treatment History of poor compliance		Lack of access to dependable communication (i.e., phone, email) Lack of access to mental health and/or addiction care specialist for continued follow-up care	

Abbreviations: OPAT, outpatient parenteral antibiotic therapy; SUD, substance use disorder; PICC, peripheral inserted central catheter.

## Data Availability

No new data were created or analyzed in this study. Data sharing is not applicable to this article.
